# 非变性质谱和紫外激光解离在蛋白质结构和相互作用分析中的应用

**DOI:** 10.3724/SP.J.1123.2024.01021

**Published:** 2024-07-08

**Authors:** Jieying XUE, Zheyi LIU, Fangjun WANG

**Affiliations:** 中国科学院大连化学物理研究所,中国科学院分离分析化学重点实验室,辽宁 大连 116023; CAS Key Laboratory of Separation Sciences for Analytical Chemistry, Dalian Institute of Chemical Physics, Chinese Academy of Sciences, Dalian 116023, China

**Keywords:** 非变性质谱, 紫外激光解离, 蛋白质结构, 蛋白质相互作用, 综述, native mass spectrometry (nMS), ultraviolet photodissociation (UVPD), protein structure, protein-protein interaction, review

## Abstract

蛋白质结构及相互作用的动态变化与其生物学功能密切相关,对蛋白质结构及相互作用进行精准探测和分析面临着巨大挑战。非变性质谱(nMS)是一种能够在近生理条件下将蛋白质及其复合物通过电喷雾离子化引入气相离子并进行质谱分析的方法。通过直接测定溶液中蛋白质及其复合物的组成或整合多种质谱解离技术,nMS可获取蛋白质及其复合物的计量关系、组装形式、解离常数、构象变化、结合界面及作用位点等关键信息,以揭示蛋白质相互作用与生物学功能之间的关系。紫外激光解离(UVPD)技术,特别是采用了193 nm准分子激光的UVPD是近年来迅速发展起来的质谱解离技术,其可以高效解离非变性蛋白质骨架,并保留碎片离子中的氢键等非共价相互作用力,从而实现单氨基酸位点分辨的蛋白质动态结构和相互作用质谱解析。本综述主要介绍了nMS和UVPD技术在蛋白质动态结构和相互作用分析中的应用和最新进展,包括由位点突变及配体结合等引起的蛋白质动态结构和相互作用变化,最后对蛋白质nMS表征的未来发展方向做出了展望。

## Abstract (en)

Dynamic changes in the structures and interactions of proteins are closely correlated with their biological functions. However, the precise detection and analysis of these molecules are challenging. Native mass spectrometry (nMS) introduces proteins or protein complexes into the gas phase by electrospray ionization, and then performs MS analysis under near-physiological conditions that preserve the folded state of proteins and their complexes in solution. nMS can provide information on stoichiometry, assembly, and dissociation constants by directly determining the relative molecular masses of protein complexes through high-resolution MS. It can also integrate various MS dissociation technologies, such as collision-induced dissociation (CID), surface-induced dissociation (SID), and ultraviolet photodissociation (UVPD), to analyze the conformational changes, binding interfaces, and active sites of protein complexes, thereby revealing the relationship between their interactions and biological functions. UVPD, especially 193 nm excimer laser UVPD, is a rapidly evolving MS dissociation method that can directly dissociate the covalent bonds of protein backbones with a single pulse. It can generate different types of fragment ions, while preserving noncovalent interactions such as hydrogen bonds within these ions, thereby enabling the MS analysis of protein structures with single-amino-acid-site resolution. This review outlines the applications and recent progress of nMS and UVPD in protein dynamic structure and interaction analyses. It covers the nMS techniques used to analyze protein-small-molecule ligand interactions, the structures of membrane proteins and their complexes, and protein-protein interactions. The discussion on UVPD includes the analysis of gas-phase protein structures and interactions, as well as alterations in protein dynamic structures, and interactions resulting from mutations and ligand binding. Finally, this review describes the future development prospects for protein analysis by nMS and new-generation advanced extreme UV light sources with higher brightness and shorter pulses.

蛋白质是生命体内最基本的分子之一,参与生命体内几乎所有的生物过程,包括催化反应、信号传导和免疫反应等^[1]^。蛋白质的功能与其结构密切相关,针对蛋白质结构进行研究有助于蛋白质功能、稳定性和相互作用的预测以及药物靶点的设计等^[2]^。利用X射线晶体衍射(XRD)^[3]^、核磁共振(NMR)^[4]^和冷冻电镜(Cryo-EM)^[5]^等技术虽可以得到蛋白质的结构信息,但这些技术对样品纯度及用量的要求较高,并且利用上述技术对由位点突变和配体结合等功能调控引起的蛋白质动态结构及相互作用变化进行表征的难度较大。

质谱(MS)是目前蛋白质研究领域的重要技术手段之一,具有高精度、高灵敏度和高通量等特点^[6,7]^。结构质谱分析技术的不断进步使其成为XRD、NMR和Cryo-EM等蛋白质结构分析技术的一个重要补充工具,能够获取高度互补的结构信息^[8]^。基于MS的结构蛋白质组学分析方法包括基于化学标记的结构质谱和非变性质谱(native mass spectrometry, nMS),二者均被广泛应用于蛋白质的动态结构、相互作用及功能研究^[9⇓⇓-12]^。与基于化学标记的结构质谱不同,nMS无需将蛋白质酶解为肽段,其直接以完整蛋白质及其复合物为研究对象,能够在近生理条件下对蛋白质及其复合物的结构及相互作用进行表征,是蛋白质结构分析的新策略^[13⇓-15]^。20世纪90年代,Li等^[16]^首次利用电喷雾电离质谱(electrospray ionization mass spectrometry, ESI-MS)研究了肌红蛋白、血红蛋白、细胞色素C与血红素之间的相互作用,自此ESI-MS被广泛运用在蛋白质复合物的结构研究中。近年来,nMS逐渐成为蛋白质复合物结构表征的重要技术,基于nMS的蛋白质结构解析技术时间发展图见图1;将nMS与碰撞诱导解离(CID)、表面诱导解离(SID)、紫外激光解离(UVPD)等技术相结合,能够获得蛋白质复合物的组成、配体相互作用等更多高级结构信息。与NMR和Cryo-EM相比,nMS无需进行蛋白质同位素标记,对样品纯度和用量的要求低,能够分析相对分子质量较小的整体蛋白质和大分子蛋白质组装体;但nMS对缓冲液浓度和pH等条件有严格要求,在对疏水性膜蛋白结构进行解析时存在一定的挑战。利用nMS可获得蛋白质及其复合物的相对分子质量、复合物的组成和化学计量关系、组装形式、解离常数等信息^[17⇓⇓⇓⇓⇓⇓⇓-25]^。通过整合多种质谱解离模式,nMS在维持蛋白质结构不变的同时可对非变性蛋白质离子的骨架进行高效解离,从而实现蛋白质结构的精细质谱解析^[26,27]^。传统的碰撞解离模式(如CID和高能碰撞解离(HCD))会优先破坏非变性蛋白质内部的非共价相互作用,引发蛋白质结构解聚,蛋白质骨架难以解离,序列覆盖度较低^[28]^。

**图1:**
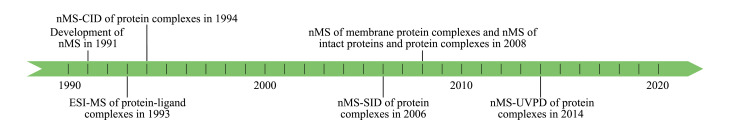
基于nMS的蛋白质结构解析技术时间发展图

此外,在非变性蛋白质离子的解离方面,基于电子的质谱解离模式(如电子转移解离(ETD))同样面临很多问题,其虽能够保留非共价相互作用,但碎片离子的产率低,主要产生未解离的减电荷物种^[29]^。UVPD是近年来发展迅速的一种基于高能紫外光子的快速质谱解离技术,其能够实现蛋白质序列的高覆盖度解离,并保留非共价相互作用信息,在蛋白质结构分析领域展现出了广阔的应用前景^[29,30]^。与NMR和Cryo-EM相比,UVPD能够实现由位点突变、配体结合等功能调控引起的蛋白质动态结构及相互作用变化的质谱解析,但针对大分子蛋白质和蛋白质复合物的结构解析,UVPD仍面临挑战。将不同波长(157、193、213、266和355 nm等)紫外激光应用于生物分子的UVPD研究已有相关报道,其中蛋白质骨架中的酰胺键对193 nm紫外激光具有强吸收,同时193 nm紫外激光可在空气中进行传播,因此目前基于193 nm紫外激光的应用最为广泛^[31⇓⇓⇓⇓-36]^。本综述主要介绍近年来nMS和UVPD技术在蛋白质动态结构和相互作用分析中的应用,并对蛋白质nMS表征的未来发展方向进行展望。

## 1 nMS在蛋白质结构和相互作用分析中的应用

nMS中的“native”仅代表蛋白质及其复合物溶液进入MS之前的状态。在进入MS前,样品通常溶解在近生理条件下的挥发性缓冲液中(如乙酸铵缓冲液(pH 7.0)),以保持蛋白质及其复合物在溶液中的折叠状态^[37]^。作为无化学标记的结构蛋白质组学分析方法,nMS主要采用纳米电喷雾离子化(nano-ESI)软电离技术将非变性溶液中的完整蛋白质及其复合物传递至MS中;同时,根据蛋白质及蛋白质复合物的理化性质来优化nano-ESI条件,有助于维持蛋白质在气相中的紧凑三级和四级结构^[37,38]^。综上,nMS所具备的非变性条件使其非常适用于蛋白质及蛋白质复合物的结构和相互作用分析。

### 1.1 蛋白质与小分子配体之间的相互作用分析

蛋白质与小分子配体之间相互作用所导致的构象变化通常会对蛋白质的功能产生影响,因此对蛋白质与小分子配体之间的相互作用进行研究有助于阐明各种生理过程的分子机制^[39,40]^。Root等^[41]^采用非变性ESI-MS对拟南芥中类异戊二烯生物合成酶(*At*IspF)与各种配体(金属辅助因子Zn^2+^、底物分子和合成抑制剂等)之间的相互作用进行了表征,如图2所示。基于MS数据,Root等阐明了芳基磺胺类抑制剂对*At*IspF的抑制机制,包括芳基磺胺类抑制剂与底物竞争结合口袋的竞争性抑制机制以及从*At*IspF活性位点中提取Zn^2+^而产生的非竞争性抑制机制,其中后者对*At*IspF活性的抑制作用更加明显。Yu等^[18]^利用nMS确定了核苷酸与蛋白酶体激活核苷酸酶(proteasome-activating nucleotidase, PAN)六聚体结合时的化学计量关系,并发现与PAN六聚体紧密结合的6个二磷酸腺苷(adenosine diphosphate, ADP)分子可以被替换为ADP类似物和三磷酸腺苷(adenosine triphosphate, ATP)类似物;同时,PAN的突变体PAN^KA^不能与ADP类似物产生相互作用,但却可以与ATP类似物产生相互作用。

**图2:**
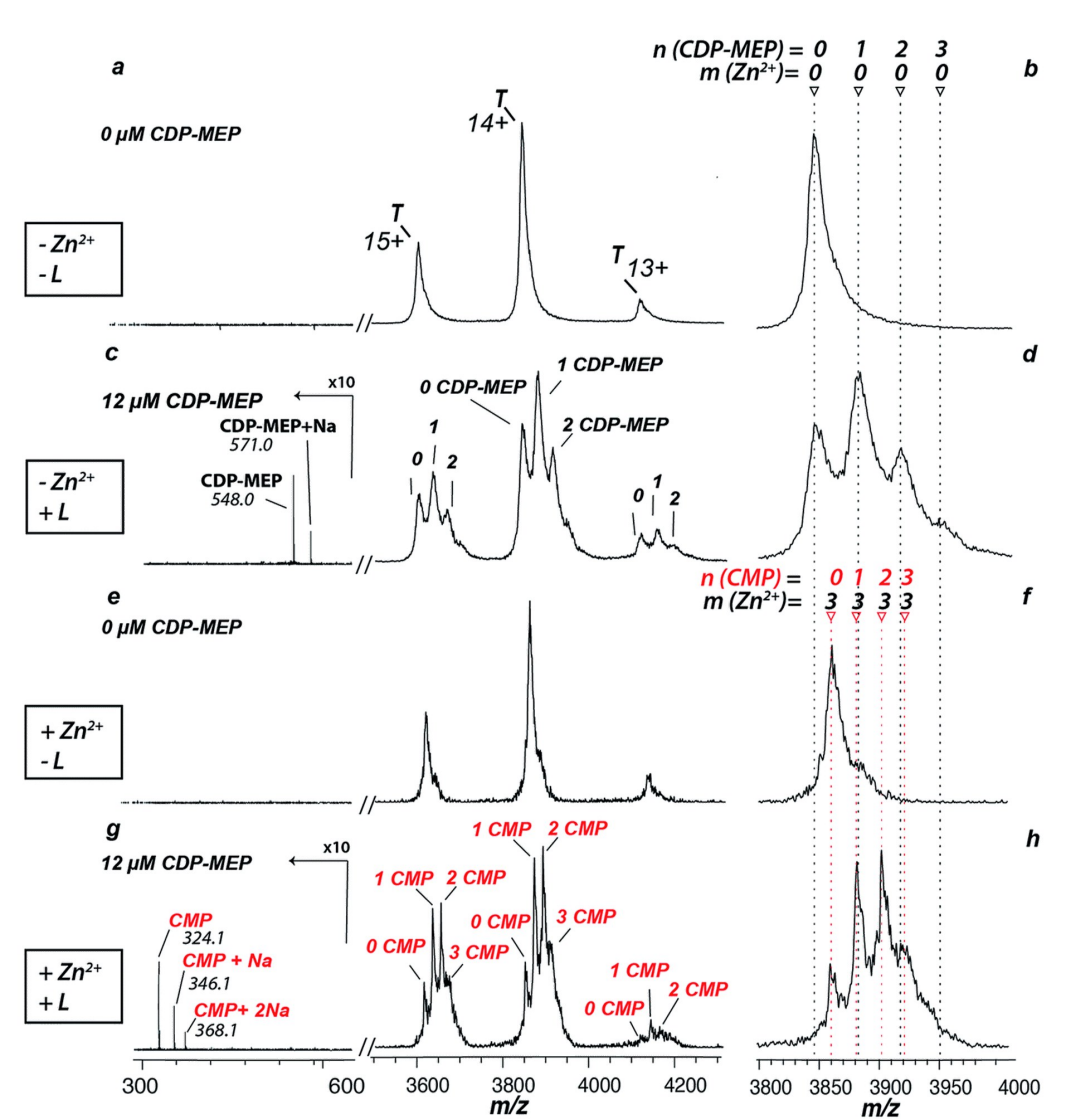
*At*IspF与4-二磷酸核糖醇2-C-甲基-D-赤藓糖醇2-磷酸(CDP-MEP)、Zn^2+^结合的非变性ESI-MS图^[41]^

El-Baba等^[39]^利用nMS探究了新冠病毒(SARS-CoV-2)中主蛋白酶(main protease, M^pro^)的变构抑制作用,研究表明,结合在二聚体界面的小分子1-甲基-*N*-{[(2*S*)-氧杂环戊烷-2-基]甲基}-1*H*-吡唑-3-甲酰胺(x1187)与结合在溶剂暴露表面的2-{[(1*H*-苯并咪唑-2-基)氨基]甲基}苯酚(x0390)、(5*S*)-7-(吡嗪-2-基)-2-氧-7-氮杂螺[4.4]壬烷(x0425)和(2*R*,3*R*)-1-苄基-2-甲基哌啶-3-醇(x0464)都可以通过变构调控来抑制底物裂解;实验还发现,结合在二聚体界面的小分子x1187能够破坏M^pro^二聚体的稳定性。Beveridge等^[42]^利用nMS确定了蛋白水解靶向嵌合体(proteolysis targeting chimera, PROTAC)对不同蛋白质的特异性结合差异,除了优先形成的三元复合物(泛素E3连接酶-PROTAC-目标蛋白质),实验还检测到了各种中间物种。由此可见,nMS可用于直接比较潜在底物和各种PROTAC的结合效果,以获得最有效的降解系统。蛋白质与小分子配体之间的相互作用是药物筛选过程中的关键步骤,nMS因其能够快速、直接地表征蛋白质-小分子相互作用而被广泛用于药物筛选领域^[43,44]^。Nguyen等^[44]^将非靶向代谢组学与多步、高分辨率的nMS相结合,在5种富含黄酮类化合物的植物粗提物中鉴定到了11个可与人碳酸酐酶Ⅰ结合的配体,此外该研究还发现了先前未曾报道过的牛碳酸酐酶Ⅱ和溶菌酶的天然产物配体。

### 1.2 膜蛋白及其复合物的结构分析

膜蛋白位于磷脂双分子层的不同位置上,在细胞活动中起着至关重要的作用,目前在美国食品药品监督管理局批准的药物中,60%以上的药物靶点是膜蛋白^[45]^。近年来,nMS已成为一种多功能、高灵敏度的膜蛋白及其复合物研究技术^[46,47]^。对于膜蛋白复合物,常采用能够与nMS兼容的膜类似物(如去垢剂、纳米盘等)来增加生物相容性,并通过质谱仪所配备的激活方式(如气体碰撞激活、红外光激活)从膜类似物中释放出离子^[48⇓-50]^。Yen等^[51]^利用nMS研究了由不同配体引发的β1-肾上腺素能受体(β1AR)对G_s_和G_i/o_蛋白偶联的偏向性,并结合氢氘交换质谱(HDX-MS)检测结果发现,异丙肾上腺素能够增强细胞内环3(ICL3)的结构动态性,推测ICL3对偏向性信号的传导具有关键作用。此外,该实验还证明了内源性锌离子能够稳定β1AR-G_s_蛋白质复合物,并结合分子动力学模拟及突变实验提出了锌离子在β1AR-G_s_蛋白质复合物形成过程中的别构调控机制。Lutomski等^[52]^利用改进的Orbitrap Eclipse Tribrid质谱仪,将10.6 μm红外激光聚焦到双四极杆线性离子阱的高压池中,使受到电离的蛋白质复合物转移至高压池中。通过调节红外激光的输出功率,该方法可以有效去除蛋白质复合物中的去垢剂,释放出非变性膜蛋白和保留非共价相互作用的膜蛋白复合物,从而获得氨通道(AmtB)与脂质的结合情况(图3);此外,通过增加激光输出功率还能得到非变性膜蛋白的序列信息。为了避免去垢剂和其他化学试剂可能产生的伪影,Robinson课题组^[53]^开发了一种无需使用去垢剂或其他化学试剂的膜蛋白研究方法,即将经过超声处理后的膜囊泡进行膜蛋白复合物质谱分析(SoLVe-MS),并与组学策略相结合,获得了亚基的化学计量比以及膜蛋白与辅助因子、脂质之间的相互作用。

**图3:**
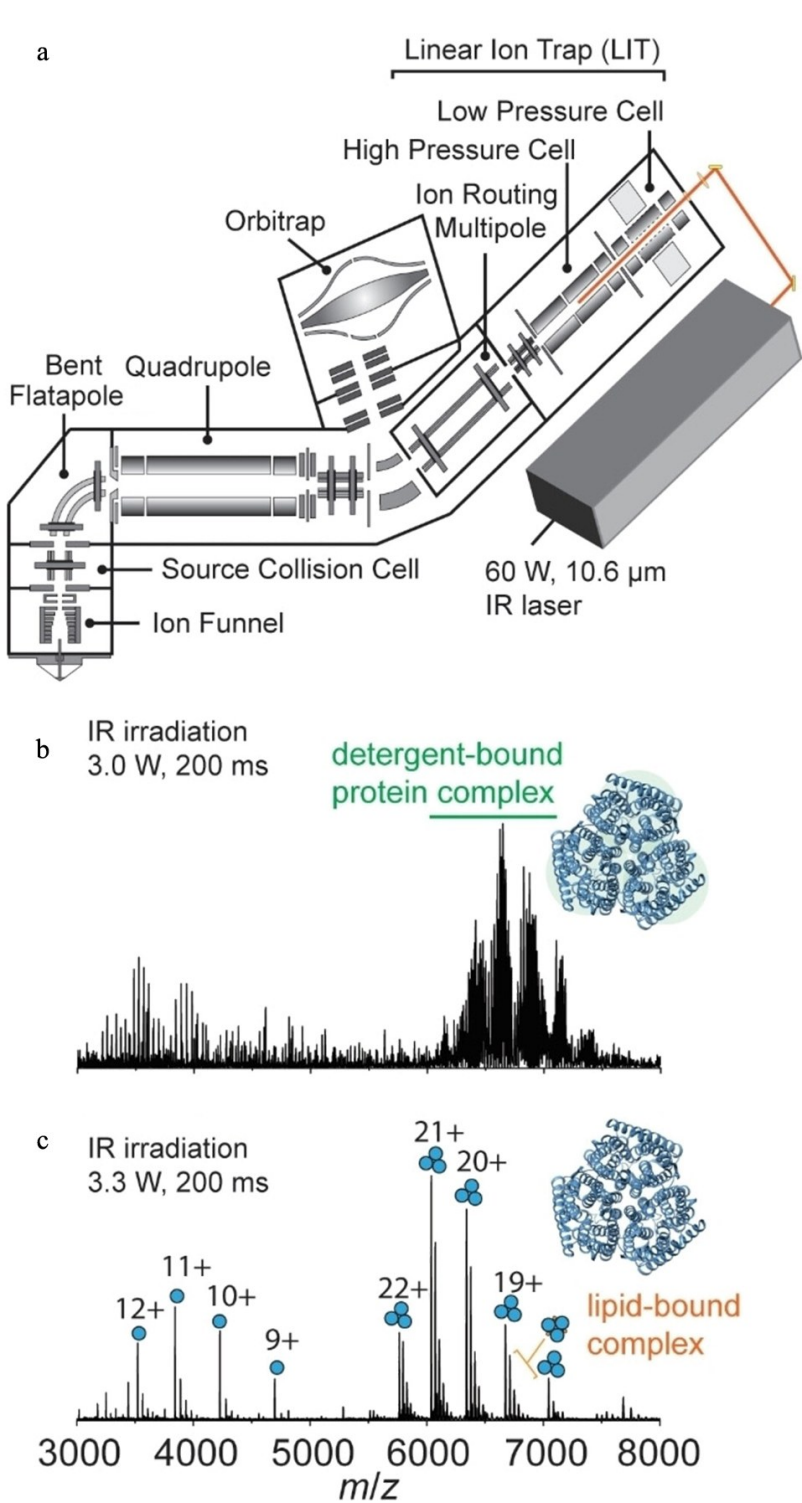
(a)改进的Orbitrap Eclipse Tribrid质谱仪示意图和(b,c)AmtB在不同功率红外激光下的nMS图^[52]^

### 1.3 蛋白质-蛋白质相互作用(PPIs)分析

PPIs对于维持细胞稳定所需的生理活动至关重要,对PPIs进行分析有助于理解PPIs的生理功能和阐明疾病发作的机制,从而为治疗疾病和药物研发提供新策略^[54]^。本课题组^[20]^利用nMS对细胞周期蛋白依赖性激酶12、13(CDK12、CDK13)与细胞周期蛋白K(CycK)复合物(CDK12/CDK13-CycK)在多种小分子抑制剂调控下的动态构象变化进行了检测,结果发现,抑制剂SR-4835能够特异性地减弱CDK12/CDK13与CycK之间的相互作用,并通过变构调节来诱导CDK12/CDK13-CycK的解离(图4)。Cruz等^[55]^利用nMS和高速原子力显微镜(HS-AFM)对疫苗候选物葡萄球菌蛋白A(SpA)与免疫球蛋白G(IgG)之间的相互作用进行了研究,结果表明,SpA可以通过竞争性结合IgG单体上恒定区之间的相互作用区域来特异性阻断IgG六聚体化以及下游补体的激活。

**图4:**
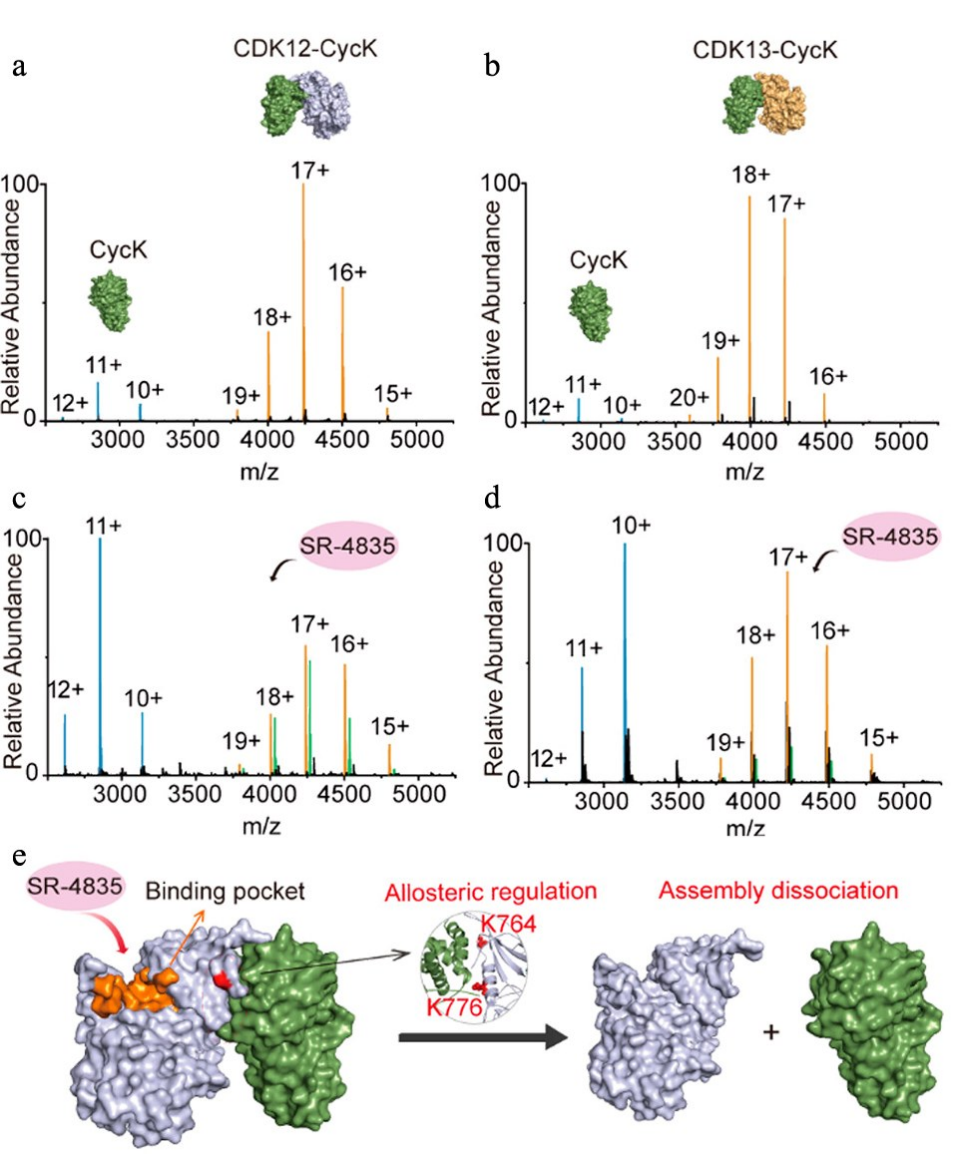
CDK12/CDK13-CycK蛋白质复合物在有、无抑制剂SR-4835条件下的nMS图及SR-4835诱导的CDK12/CDK13-CycK蛋白质复合物解离示意图^[20]^

罗宇翔等^[56]^采用nMS检测了切除*N*-糖前后,新冠病毒刺突蛋白受体结合区(RBD)与受体血管紧张素转化酶2(ACE2)所形成复合物的稳定性;结果表明,RBD中*N*-糖的切除会减弱RBD与ACE2之间的相互作用。nMS还可以用于铁硫簇桥连蛋白复合物的研究,Jia等^[57]^将快速在线缓冲液置换(OBE)与nMS结合,对谷氧还蛋白5(GLRX5)与无配体铁氧还蛋白2(FDX2)之间的铁硫簇转移反应进行研究,并提出了铁硫簇转移过程的新机制:在铁硫簇转移过程中,同源二聚体GLRX5中的铁-半胱氨酸键发生裂解,释放出两个谷胱甘肽分子,并在FDX2受体中形成新的铁-半胱氨酸键。

nMS虽能够在近生理状态下直接探测蛋白质复合物的相对分子质量,获得亚基组成、化学计量比、PPIs以及蛋白质-配体相互作用等信息^[18,21,41]^,但nMS所能提供的蛋白质动态构象变化和配体结合位点等信息有限,因此需要对蛋白质进行解离,并捕捉碎片离子以获得更多的二级、三级和四级结构信息。

## 2 UVPD在蛋白质动态结构和相互作用分析中的应用

当非变性蛋白质离子在维持其内部非共价相互作用不变的前提下发生骨架解离时,所产生的碎片离子与其三维结构有关;通过对碎片离子进行检测和鉴定,可以实现单氨基酸位点分辨的蛋白质结构质谱分析。Li等^[58]^将不同质谱解离模式应用于蛋白质复合物离子的结构解析中,基于碎片离子信息可获取翻译后修饰、位点突变和蛋白质形态等信息。电子捕获解离(ECD)技术对蛋白质结构具有选择性,可揭示蛋白质复合物界面及表面的活性氨基酸位点,但通过该技术所获得的整体蛋白质序列剪切率低。在高通量蛋白质组学背景下,基于光激活的UVPD因具有超快的解离速度、高能量沉积、保留修饰和高序列覆盖度等特点而备受关注^[59,60]^。目前普遍认可的UVPD机制如下:首先蛋白质分子与紫外光之间会产生相互作用,导致蛋白质分子中的电子受到激发,从基态跃迁至激发态;处于激发态的电子主要通过两个途径产生碎片离子,即直接解离和内部转换与分子内振动能量再分配(IVR)。UVPD通常倾向于非选择性地解离蛋白质主链,断裂蛋白质中的大部分化学键,与其他解离方式相比,UVPD能够提供更高的序列覆盖度^[29,61]^。

2014年,Brodbelt课题组^[29]^详细对比了非变性血红素/肌红蛋白复合物的CID、HCD、ETD和UVPD质谱图,发现CID和HCD主要导致血红素的释放,ETD主要产生未解离的减电荷离子,而UVPD可以保留含血红素的碎片离子,并实现血红素/肌红蛋白复合物的高覆盖度解离(图5)。UVPD-MS可以提供高序列剪切率,并能产生保留了非共价相互作用的碎片离子,以用于定位配体结合区域或位点以及研究单氨基酸突变、配体结合对蛋白质结构和功能的影响^[38,40,62⇓⇓⇓⇓⇓-68]^。将UVPD与nMS结合,可以在维持非变性蛋白质离子内部非共价相互作用不变的前提下,对进入质谱的非变性蛋白质离子进行紫外激光解离。在UVPD过程中,蛋白质主链的断裂抑制或增强取决于不同区域的灵活性或可及性以及这些区域是否被配体屏蔽或参与其他稳定的分子相互作用;这些相互作用能够稳定蛋白质复合物结合区域位点的微观结构,并导致紫外激光解离难度的增加以及光解离碎片与复合物之间的分离受到阻碍,进而使UVPD过程中蛋白质复合物结合区域位点的解离效率降低;因此,通过分析位点碎片的产率可以解析蛋白质复合物的结合区域或位点^[38,63,68]^。

**图5:**
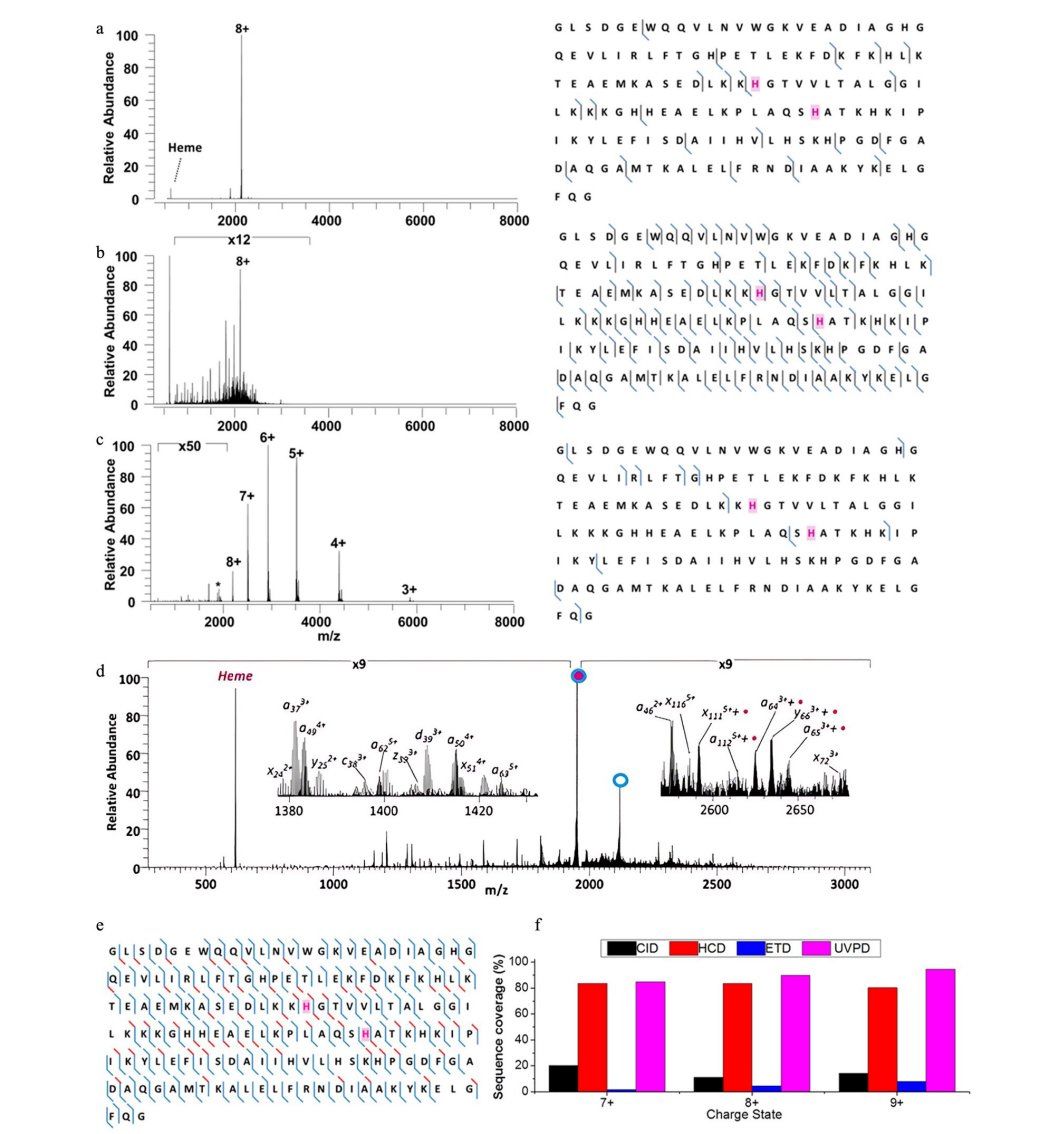
非变性血红素/肌红蛋白复合物的(a)CID、(b)HCD、(c)ETD、(d,e)UVPD二级质谱图及相应的解离序列匹配结果;(f)不同解离模式下所获得的序列覆盖度^[29]^

### 2.1 气相蛋白质的结构和相互作用分析

通过离子迁移质谱(IMS)虽可以获得气相蛋白质碰撞截面积的变化,但对于分析分子内溶剂化发生的具体位置和引起蛋白质结构变化的分子机制等方面,IMS仍受到很多限制^[69]^。本课题组^[38]^利用UVPD探测到肌红蛋白带电残基侧链分子内溶剂化的具体位点,并阐明了其对蛋白质结构影响的分子机制。研究发现,在高电荷价态下,蛋白质的气相结构易受分子内溶剂化效应的影响而偏离溶液态结构,而在蛋白质高电荷密度区域络合的冠醚可以避免带电侧链的分子内溶剂化,使得蛋白质离子的气相结构更加接近溶液状态(图6)。Bonner等^[70]^报道了一种新的分子内过程,即光诱导的电子转移解离(PETD),并通过PETD方法检测到了多肽和蛋白质中两性离子对或盐桥的存在,该实验结果表明,通过理论计算模拟气相蛋白质结构时,不仅要考虑包含盐桥的电荷态异构体,还要考虑非碱性残基上的质子化作用。Zhou等^[67]^利用UVPD技术,在不同程度的源内激活条件下对酒精脱氢酶的气相结构变化进行观察,所得研究结果证实了蛋白质复合物的电荷导向去折叠机制。

**图6:**
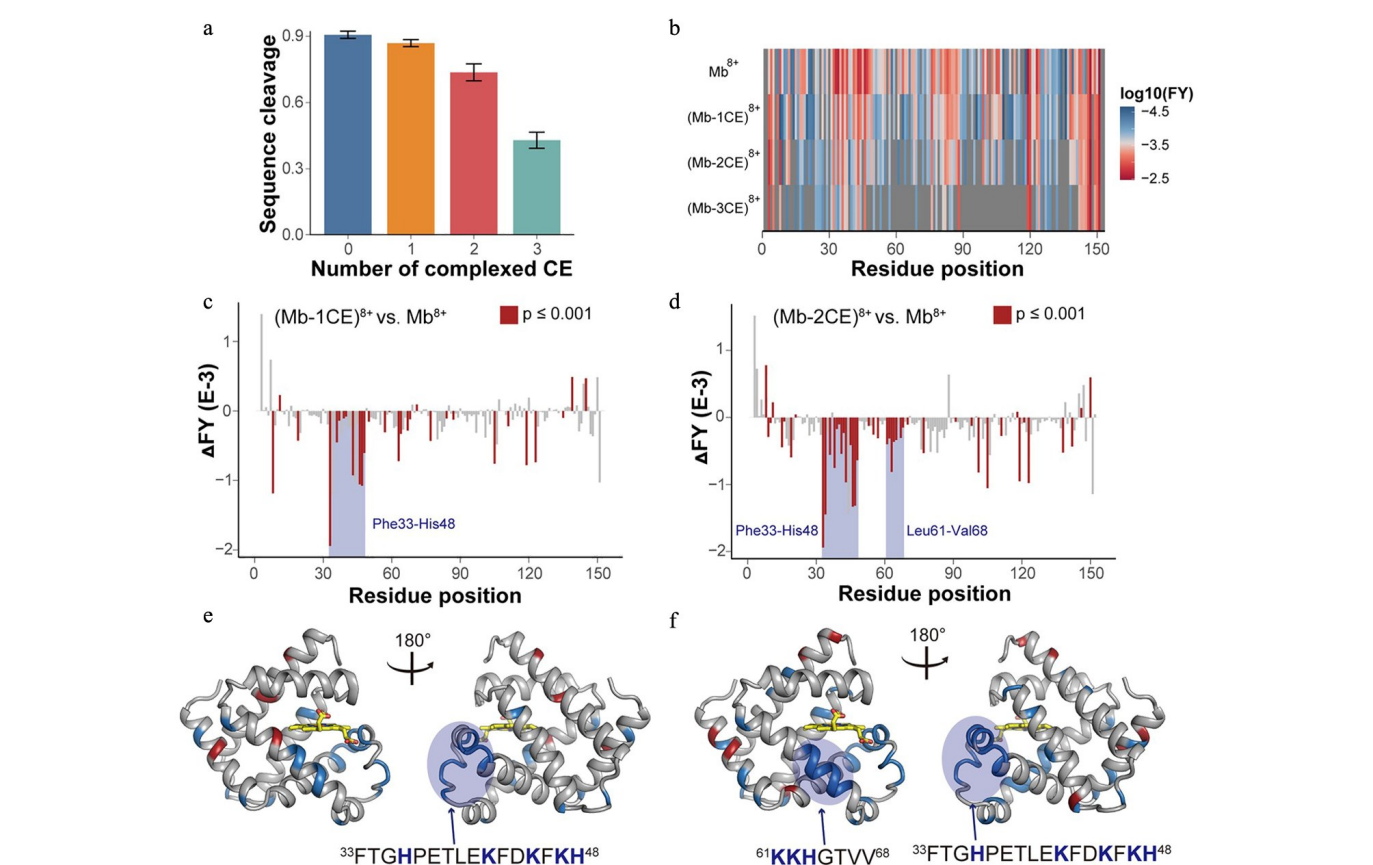
肌红蛋白络合冠醚的UVPD分析^[38]^

### 2.2 单氨基酸突变的动态结构分析

单氨基酸突变,尤其是蛋白质上催化位点、结合口袋和蛋白质-蛋白质界面的突变,可能会导致蛋白质的变构、蛋白质整体稳定性的降低以及蛋白质功能的受损,甚至会引起相关疾病^[71⇓-73]^。对蛋白质的结构变化进行研究有助于了解疾病的发生机制,并开发出针对相关疾病的治疗方法。

致病性大肠杆菌是引起尿路感染的主要原因之一,其对用于抑制二氢叶酸还原酶(DHFR)活性的抗生素甲氧苄啶(TMP)具有明显的耐药性,并且大肠杆菌的耐药性可通过调节DHFR中的单个氨基酸突变来获得^[74]^。Cammarata等^[75]^通过UVPD-nMS对DHFR的两个临床突变体P21L和W30R进行了研究(图7);结果表明,与野生型DHFR相比,P21L突变体的UVPD碎片产率有轻微变化,而W30R突变体在底物/抑制剂结合口袋和M20环区域的UVPD碎片产率有明显变化。研究发现,W30R和P21L会分别通过两种独特机制来诱导DHFR产生结构变化,一是直接调节蛋白质的底物结合区,二是调节M20环的刚性,并最终使致病性大肠杆菌获得耐药性。Mehaffey等^[32]^将碰撞激活与193 nm UVPD结合,对支链氨基酸转氨酶2(BCAT2)二聚体及其单氨基酸变体T186R进行研究。研究中不仅识别到了辅助因子吡哆醛磷酸盐(PLP)的结合位点,还发现了单氨基酸突变会导致蛋白质与PLP的结合作用不稳定。此外,UVPD-nMS还可以用于研究单氨基酸突变对下游信号的影响。例如,Brodbelt课题组^[68]^研究了与不同配体(二磷酸鸟苷、三磷酸鸟苷类似物)结合后,Kirsten大鼠肉瘤病毒癌基因同源物(K-Ras)编码蛋白的3个G12X突变(G12C、G12V、G12S)与下游效应蛋白Raf异源二聚化的影响。研究结果表明,不同K-Ras突变通过稳定或破坏Raf的相互作用界面(α, β界面)来调节下游信号传导,从而影响癌症的发展,该研究为解释不同K-Ras突变在下游信号传导中的复杂机制方面提供了新的见解。

**图7:**
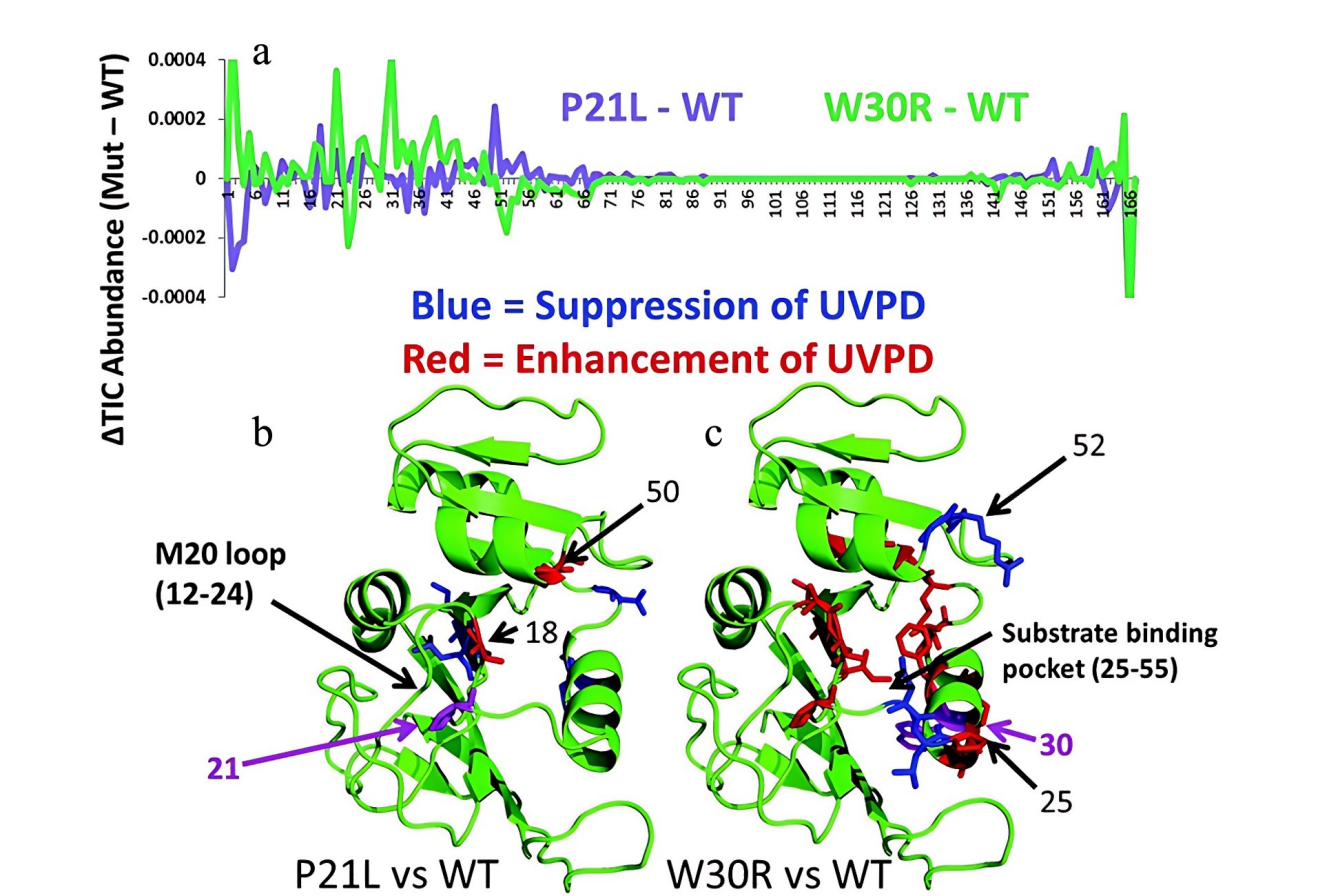
UVPD-nMS用于解析由DHFR突变体(P21L和W30R)引起的DHFR动态结构变化^[75]^

### 2.3 蛋白质与小分子配体之间的相互作用分析

蛋白质与小分子配体的结合可以调节蛋白质的构象和功能,从而影响细胞的酶催化、信号传导和调控等关键生物过程^[39,76,77]^。对蛋白质与小分子配体之间的相互作用进行分析有助于理解生物体内的生物过程,为药物设计和疾病治疗提供重要指导。UVPD的高能量沉积和快速碎裂过程能够使蛋白质与配体之间的非共价相互作用在共价键被断裂的同时得到保留,这使得UVPD非常适用于蛋白质复合物的表征^[40,60,63,78⇓-80]^。

免疫共受体(如CD28)是癌症治疗中常见的免疫治疗靶点,其通过酪氨酸磷酸化(pY)激活并诱导下游的信号蛋白形成蛋白质复合物,进一步激活T细胞^[81]^。Chen等^[63]^合成了一系列不同pY修饰的CD28,并发现CD28的pY218与蛋白激酶Cθ(protein kinase Cθ, PKCθ)的C2结构域能够发生特异性结合;同时本课题组^[63]^利用nMS和193 nm的UVPD研究了二者的结合机制(图8)。UVPD能够识别出C2与pY218结合的3个结合区域和3个核心位点,且区域2是文献已报道过的pY结合口袋^[82]^,区域1和区域3分别位于结合口袋的两侧。实验结果说明,除与配体结合之外的残基在蛋白质复合物的形成过程中也起着重要作用,其可能通过长程的变构相互作用效应来影响C2的结构和功能。Hernandez-Alba等^[83]^利用HCD、ETD和213 nm的UVPD对药物抗体比值为4的第三代特异性抗体偶联药物(ADC)进行了自中而下的质谱分析。结果表明,UVPD的整体序列覆盖率高于HCD和ETD,并且可以生成更多含有药物结合位点或糖基化位点信息的碎片离子,从而能够更加准确地鉴定药物偶联和糖基化结合位点。蛋白质与金属离子的结合在生物体内发挥着重要的生理作用,涉及酶催化、氧气运输、电子传递、信号传导和结构稳定等方面^[23,84]^。Crittenden等^[85]^利用UVPD对3种能够与金属离子结合的蛋白质体系进行研究;结果表明,采用UVPD技术所获得的结合区域与XRD和Cryo-EM结果一致,证明了UVPD在确定和分析各类蛋白质金属结合区域方面的实用性。UVPD-nMS还可以用于检测磷酸转移酶腺苷酸激酶(AK)在酶循环过程中的动态结构变化。研究发现,辅助因子镁离子的存在或缺失都会引起AK中与磷酸基团相互作用的两个环区域发生构象变化。上述研究结果证实,镁离子能够加速腺苷酸结合区域和ATP结合区域的打开过程^[40]^。除此之外,UVPD(激发光波长≤150 nm)也可用于蛋白质-配体复合物的表征,如Canon等^[86]^采用同步辐射不同能量的光子对一种人类内在紊乱蛋白(称为IB5)及其与单宁结合形成的复合物进行解离。结果发现,当单光子能量为13.2 eV和16 eV时,均可以获得丰富的碎片离子;同时该研究成功测定了单宁配体在IB5上的结合位点。

**图8:**
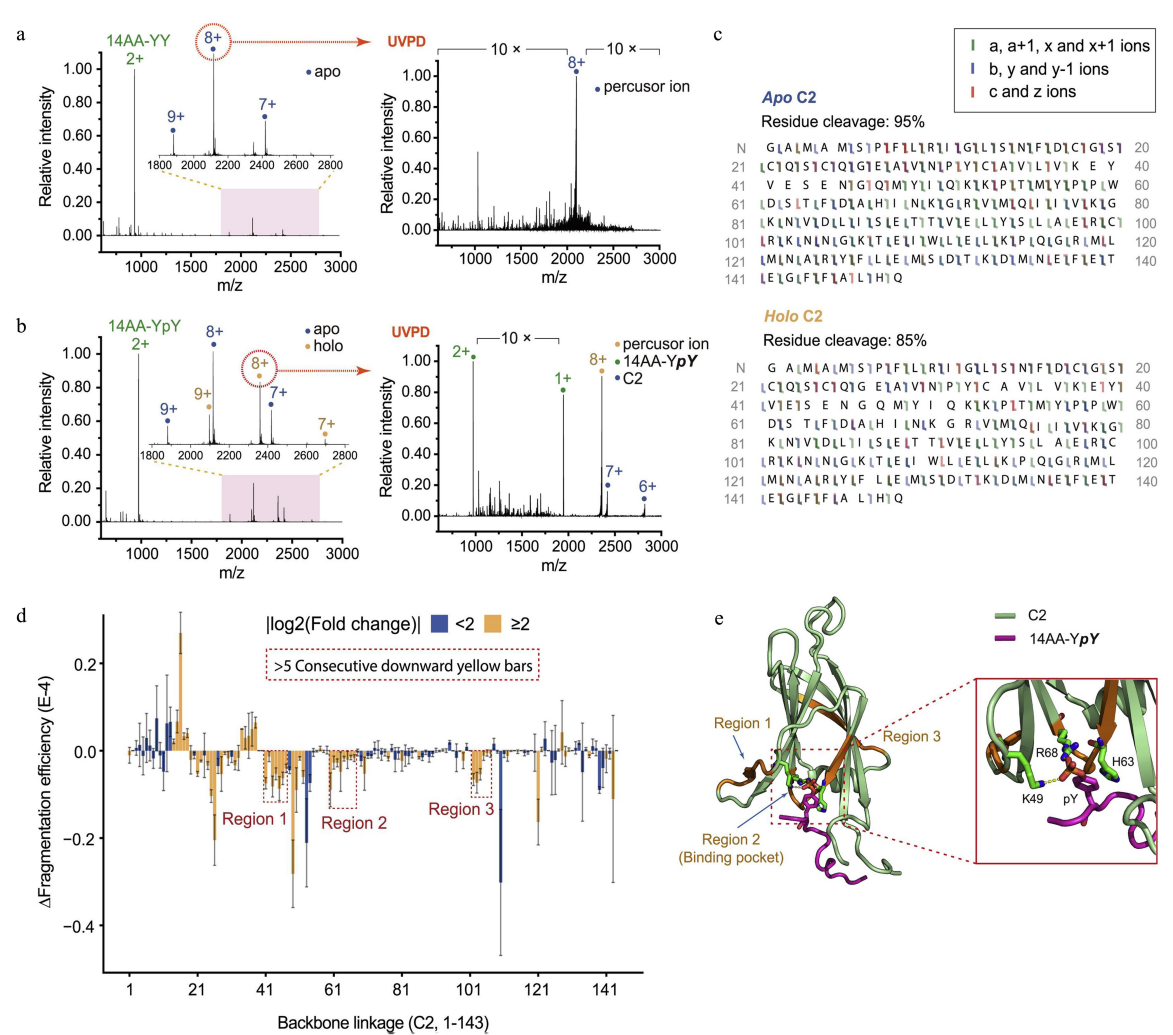
蛋白激酶Cθ的C2结构域与pY218之间相互作用的UVPD-nMS分析^[63]^

### 2.4 大分子蛋白质复合物的结构及相互作用分析

基于电子的解离技术(如ECD、ETD)和SID已被用于多聚蛋白质复合物的表征,UVPD可作为一种新的多聚蛋白质和蛋白质组装解离方法,用于获取蛋白质动态结构和相互作用等信息^[87⇓⇓⇓-91]^。Sipe等^[91]^将nMS、UVPD和碰撞诱导去折叠技术(CIU)结合,对Tautomerase蛋白质(TSF)的三聚体结构进行表征,以快速区分L2对称三聚体、F4不对称三聚体和R7不对称三聚体;基于碎片离子产率、电荷位置迁移和氢消除监测(HEM)结果,该实验推测出,亚基间的盐桥是使气相中不对称三聚体结构更稳定的主要因素。Mehaffey等^[92]^通过比较血凝素(HA)和HA-抗体复合物的UVPD碎片离子丰度,确定了抑制抗原骨架断裂的区域,并揭示了未知表位。此外,通过观察UVPD产生的碎片离子类型可以发现,在抗体-抗原复合物的串联质谱分析过程中,仅在抗原决定簇上出现了多样性的损失。Greisch等^[93]^利用UVPD技术,不仅诱导CRISPR-Cas Csy核糖核蛋白复合物中的亚基产生了丰富的碎片离子,还获得了捕光复合物B-藻红蛋白亚基与色素的结合位点信息及病毒样颗粒AaLS的序列修饰信息。Brodbelt课题组^[94]^利用nMS和UVPD对噬菌体T4基因蛋白32(T4 gp32)与单链DNA(single-stranded DNA, ssDNA)之间的非共价相互作用进行了研究(图9)。结果表明,T4 gp32的整个中心结构区域与ssDNA的底物(dT12)之间存在较强的相互作用,并且在T4 gp32的N端和C端都能检测到可以与dT12发生相互作用的区域和位点。除了上述应用外,UVPD还可以用于分析传统晶体学技术无法获得的蛋白质无序区域。

**图9:**
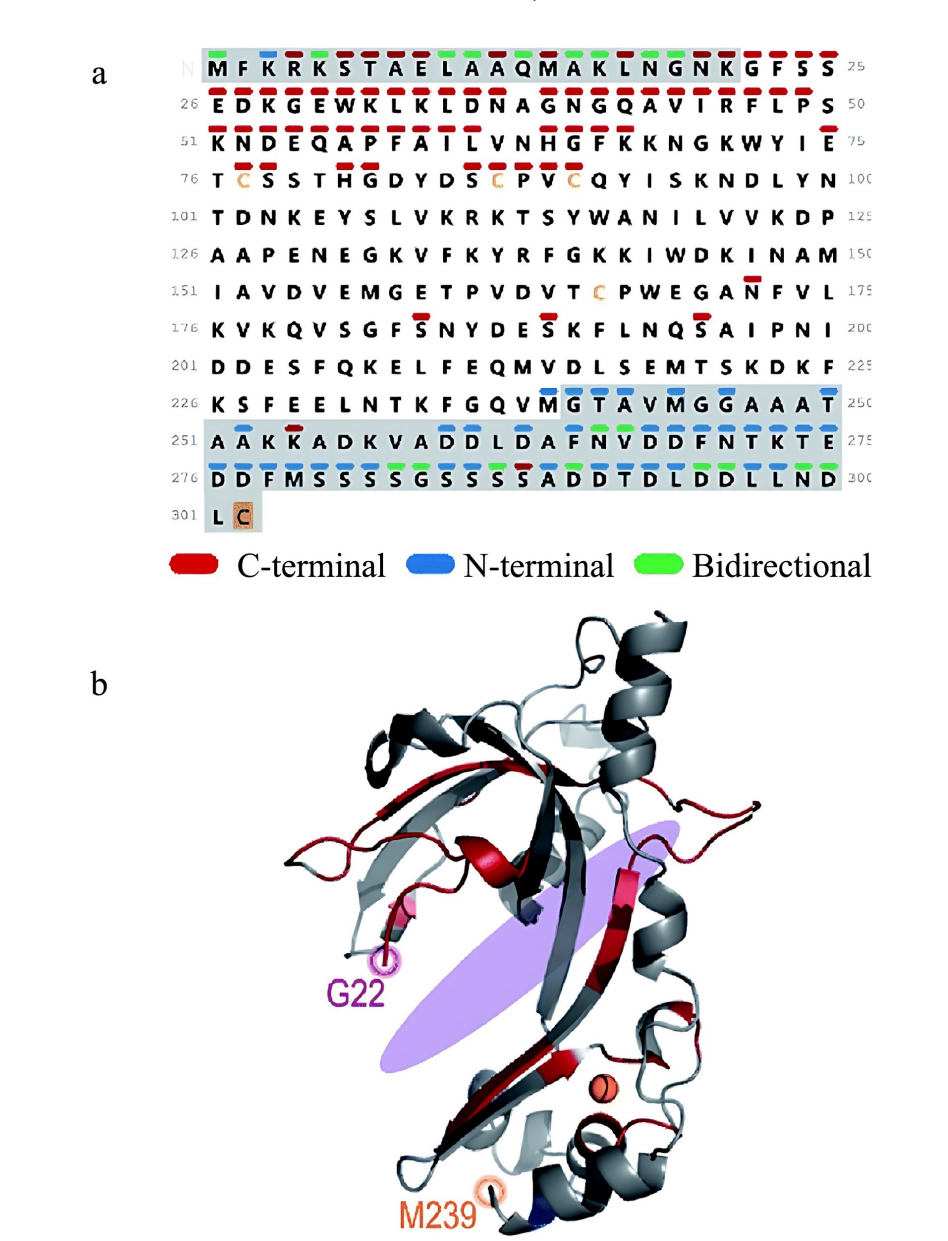
nMS和UVPD用于T4 gp32-ssDNA复合物结构的表征^[94]^

UVPD-nMS为非变性蛋白质动态结构和相互作用的解析提供了有力工具,除了对单氨基酸位点分辨的蛋白质动态结构、蛋白质和配体相互作用的表征,UVPD-nMS还有望借助于质谱仪器、数据处理软件的改进以及先进紫外光源的发展,用于大分子蛋白质复合物的结构解析,这将进一步提高科研人员对蛋白质精细结构的了解,并实现更好的靶向药物设计。黄光明课题组^[95]^提出了一种诱导电喷雾原位细胞质谱策略(“in-cell” MS),实现了细胞内蛋白质及其复合物的直接进样分析。将“in-cell”MS与UVPD结合,有望实现哺乳动物细胞中蛋白质及其复合物的原位动态结构和相互作用表征,同时这依赖于活细胞中蛋白质在线提取、分离技术和电喷雾离子化技术的改进。

## 3 总结与展望

膜蛋白是现阶段最主要的药物靶点,因此对膜蛋白的结构进行研究有助于揭示治疗药物的新靶点。nMS虽可以用于研究膜蛋白与配体之间的相互作用,但不足以获得膜蛋白的动态结构变化等信息。UVPD是一种快速的解离方法,将其与nMS联用,可以提供蛋白质序列、动态结构和相互作用等信息;未来有望借助新技术、新方法的开发将UVPD用于膜蛋白与小分子药物之间相互作用的分析,进而对药物的设计和开发进行优化,提高药物疗效和安全性。但是,在对非变性大分子蛋白质复合物的动态结构和相互作用表征方面,还需进一步提高与UVPD联用的质谱检测灵敏度,以及开发出亮度更高、脉冲更短的极紫外先进光源,以获得更高的解离效率和结构分析能力。此外,当分析样品包含多个质量相似的蛋白质复合物或一组复杂的蛋白质时,直接采用UVPD-nMS来表征蛋白质亚基组成、高级结构及构象变化,仍存在一定的挑战。为实现这一目标,需要在nMS检测之前对分析样品进行非变性分离,同时这也对非变性分离和质谱技术的整合提出了更高的要求。预计未来会有更多的非变性分离与质谱联用技术被开发出来,尤其是与质谱相容性更高的色谱固定相材料,以对更加复杂的样品进行高通量分析,这将会推动非变性复杂蛋白质组结构分析的进一步发展。
